# Supplementary material for: Effects of clown visits on stress and mood in children and adolescents in psychiatric care—Protocol for a pilot study
Source: PLoS One. 2022 Feb 18;17(2):e0264012. doi: 10.1371/journal.pone.0264012 (PMC8856575; doi:10.1371/journal.pone.0264012)
Supplement: S1 File — (PDF) [file pone.0264012.s002.pdf]

Antragsformular zur Beurteilung einer geplanten wissenschaftlichen Studie durch die Ethikkommission der Universität Wien

Stand: November 2019

Die beantragte Studie ist als Beilage anzuhängen. Es kann **zusätzlich** (!) zur kurzen Beantwortung der Fragen (max. 150 Wörter) auf den sich beziehenden Teil im Antragsformular verwiesen werden.

Nichtzutreffende Punkte bitte mit t.n.z. (trifft nicht zu) beantworten.

Die Ethikkommission macht darauf aufmerksam, dass ausschließlich formal vollständige Anträge behandelt werden. Sollten noch Ergänzungen aus formalen Gründen notwendig sein bzw. Informationen fehlen (z. B. Fragebögen), kann der Antrag erst zu einem späteren Sitzungstermin behandelt werden.

1. Allgemeines

1.1. Name der Antragstellerin/des Antragstellers

Univ.-Prof. Dr. Martina Zemp

1.2. Titel der Studie

Auswirkungen von Clownbesuchen auf das Stresserleben von Kindern und Jugendlichen im psychiatrischen Kontext

1.3. Bei der vorgelegten Studie handelt es sich um:

☐ ein drittmittelfinanziertes Projekt

☐ beantragt ☐ bereits bewilligt

Fördergeber/Universitätsinterne Projektnummer/Kostenstelle (falls bereits vorhanden):

☒ ein universitätsinternes Projekt

☐ eine Dissertation (PhD-Arbeit)

Name des Betreuers/der Betreuerin:

Die/der BetreuerIn wurde über die Einreichung informiert

☐ Ja ☐ Nein

Datum der positiv absolvierten fakultätsöffentlichen Präsentation (FÖP)

☐ eine Masterarbeit/eine Diplomarbeit

Name der Betreuerin/des Betreuers:

Einreichung wurde beantragt von:

☐ Betreuerin/Betreuer ☐ Studienrechtliches Organ

1.4. Warum bzw. wofür wird ein Votum der Ethikkommission benötigt?

☐ Die Untersuchung könnte die physische oder psychische Integrität, das Recht auf Privatsphäre oder sonstige subjektive Rechte oder überwiegende Interessen von Versuchspersonen beeinträchtigen.

☐ Das Votum wird von einem Publikationsorgan verlangt.
☐ Das Votum wird von einem Fördergeber verlangt.
☒ Sonstige Gründe, nämlich:

Das Forschungsvorhaben wird in Kooperation mit dem Verein ROTE NASEN Clowndoctors und klinischen Institutionen des Gesundheitsbereiches (z.B. Kinder- und Jugendpsychiatrien) durchgeführt. Die Beurteilung durch eine Ethikkommission wird sowohl von Seiten der Einrichtungen als auch zum Schutz der Studienteilnehmenden benötigt. Das Forschungsvorhaben wird auf Verlangen der kooperierenden Einrichtungen auch auf jeweiliger Landesebene zur ethischen Begutachtung vorgelegt werden.

1.5. Wurde für die Studie bereits ein Begutachtungsverfahren durch eine Ethikkommission durchgeführt?
☐Ja ☒Nein Wenn Ja, Gutachten beilegen

2. Kurzinformationen zur geplanten Studie

2.1. Handelt es sich um eine Teilstudie innerhalb eines größeren Forschungsvorhabens? Wenn ja: bitte den Titel dieses größeren Projekts angeben.

t.n.z

2.2. Fachdisziplin

Psychologie, Klinische Psychologie des Kindes- und Jugendalters

2.3. Kurzbeschreibung der geplanten Studie (max. 500 Wörter)

Das Studienvorhaben ist eine Pilotstudie zur Messung kurz- sowie mittelfristiger Effekte von Clownbesuchen des Vereins ROTE NASEN Clowndoctors. Im Zentrum steht das Stresserleben von Kindern und Jugendlichen im stationären oder ambulanten psychiatrischen Kontext, welche in einem wöchentlichen Abstand Clownbesuche in den Institutionen erleben. In einem quasi-experimentellen Studiendesign wird das Stresserleben der ProbandInnen mittels hormoneller Marker und Fragebögen anhand eines Pre-/Posttests erhoben. Die Zielstichprobe involviert 40 Kinder und Jugendliche, welche in psychiatrischen Einrichtungen in Österreich ambulant oder stationär behandelt werden und in einem wöchentlichen Abstand an Clownbesuchen des Vereins ROTE NASEN Clowndoctors teilhaben.

Die Datenerhebung gliedert sich in zwei Phasen. In einer ersten Phase wird in vier aufeinanderfolgenden Wochen eine Pre-/Posttest Messung des Cortisol-Levels vor sowie nach dem Clownbesuch stattfinden. Mit Hilfe standardisierter Fragebögen werden zusätzlich psychologische Belastungsmaße (subjektives Stresserleben, aktuelles Unruheempfinden sowie positiv wahrgenommener Affekt durch den Clownbesuch) bei den ProbandInnen erfasst. Die Anwendung standardisierter Fragebögen in dieser Phase erfolgt papierbasiert (paper-pencil) und im Beisein der Versuchsleitung. Die Messung des Cortisol-Levels geschieht anhand von Speichelproben. Der Speichelabstrich der Kinder und Jugendlichen wird mit Hilfe einer Salivette einmal vor sowie einmal nach der Interaktion mit den Clowns erfolgen. Der/Die ProbandIn wird hierfür aufgefordert, eine Baumwollrolle ein bis zwei Minuten im Mundraum zu befeuchten. Alle erhobenen Daten werden nach der Felderhebung pseudonymisiert behandelt.
Der Clownbesuch wird durch BegegnungskünstlerInnen des Vereins ROTE NASEN Clowndoctors in herkömmlicher Art und Weise organisiert und durchgeführt. Darüber hinaus sind diese BegegnungskünstlerInnen nicht in die Datenerhebungen miteingebunden.

In einer zweiten Phase erfolgt die Testung eines mittelfristigen Effekts der Clownbesuche. In einem Abstand von drei Monaten nach der ersten Erhebungsphase werden selbige ProbandInnen gebeten, ein weiteres Fragebogenset zur Erfassung subjektiver Belastungsmaße und zu den Erinnerungen an die Clownbesuche auszufüllen.

Die Studie wird unter der Leitung des Arbeitsbereichs Klinische Psychologie des Kindes- und Jugendalters der Fakultät für Psychologie (PI: Prof. Dr. Martina Zemp) in Kooperation mit dem Arbeitsbereich Klinische Psychologie des Erwachsenenalters (Co-PI: Prof. Dr. Urs Nater) sowie dem Verein ROTE NASEN Clowndoctors (Organisation: MMag. Simone Seebacher MA) durchgeführt.

2.4. Zielsetzung der Studie (Fragestellungen, Hypothesen etc.)

Das Ziel des Forschungsvorhabens ist die Untersuchung von kurz- sowie mittelfristigen Effekten von Clownbesuchen auf das hormonelle und psychologische Stresserleben von Kindern und Jugendlichen im psychiatrischen Kontext. Es wird eine stressreduzierende Wirkung von Clownbesuchen auf die Zielgruppe erwartet, welche anhand biopsychologischer Marker gemessen wird. Des Weiteren wird von einem kumulativen Effekt der Clownbesuche ausgegangen, der nach einer Phase von drei Monaten erkennbar ist. Im Spezifischen werden folgende Hypothesen getestet:

1. Kinder und Jugendliche zeigen ein reduziertes Cortisol-Level sowie ein geringeres subjektives Stresserleben nach dem Clownbesuch (Post-Messung) im Vergleich zu davor (Pre-Testung).
2. Je häufiger die Kinder und Jugendlichen innerhalb des Erhebungszeitraums von vier Wochen an den Clownbesuchen teilnehmen, desto stärker sind die stressreduzierenden Effekte.
3. Die subjektive Einschätzung und Erinnerung der Kinder und Jugendlichen nach drei Monaten deuten auf eine anhaltende Wirkung und Präsenz der Clownbesuche hin.
4. Die subjektive Einschätzung des Behandlungspersonals deutet auf positive Effekte der Clownbesuche auf die Stimmung des Behandlungspersonals, die Atmosphäre auf der Klinikstation und das Befinden der Kinder und Jugendlichen hin.

2.5. Wissenschaftliche und gesellschaftliche Relevanz der Studie

Krankheitserfahrungen sowie Kliniksaufenthalte sind für PatientInnen und ihre Angehörigen oftmals mit Erleben von Stress, Ängsten und Anspannung verbunden. Schmerzerfahrungen, Kontrollverlust, Verwirrung und die Trennung von nahestehenden Angehörigen kann zu physischen sowie mentalen Gesundheitsbeeinträchtigungen führen (Price et al. 2016). Auch nach einer Entlassung aus dem Spitalkontext konnten Symptome negativen Stressempfindens bei Kindern festgestellt werden, darunter erhöhte Unruhe sowie ein verringertes Selbstwertgefühl (Rennick & Rashotte 2009).

Clownbesuche finden seit Jahrzehnten in Gesundheitseinrichtungen statt, bei welchen mit Hilfe von Humor, Ablenkung und Kreativität die belastende Situation umgedeutet werden kann und somit Erleichterung und Entlastung für die Betroffenen schafft. Internationale Studien haben bereits auf die Wirksamkeit von Clownbesuchen in Bezug auf unterschiedliche Outcomes hingewiesen. Es wurde gezeigt, dass die Interaktion mit Clowns vor einem invasiven medizinischen Eingriff den empfundenen Stress und die Anspannung reduzieren kann (Vagnoli et al. 2005; Dionigi et al. 2014). Ein positiver Effekt des Clown-besuchs konnte auch in Bezug auf die Schmerzerfahrung von Kindern untersucht werden, die sich besonders schmerzhaften Untersuchungen unterziehen müssen (Goldberg et al. 2014; Ben-Pazi et al.

2017). Darüber hinaus zeigen Resultate eine anspannungsverringernde Wirkung, die mit einer Sedierung vergleichbar ist (Viaggiano et al. 2015).
Vereinzelte Studien haben außerdem Auswirkungen der Clownbesuche auf das Behandlungspersonal und den Arbeitsalltag im Krankenhaussetting aufgedeckt. Die Anwesenheit von Clowns wird von AnästhesistInnen besonders vor und nach einer Operation befürwortet (Smerling et al. 1999). Ein sehr positiver Effekt wurde innerhalb des Pflegepersonals auf Kinderstationen festgestellt, bei welchen es zu Veränderungen sowohl in der Kommunikation mit PatientInnen als auch des allgemeinen Arbeitsalltags kommt (Blain et al. 2012).

Der gesundheitsfördernde Effekt von Clownbesuchen wurde bereits aus unterschiedlichen Blickwinkeln untersucht. Die Wirkung auf spezifische Zielgruppen sowie die Erhebung biopsychologischer Marker wurde bis dato jedoch nicht ausreichend erforscht. Die vorliegende Pilotstudie wird einen Beitrag dazu leisten, den Effekt von Clownbesuchen auf Kinder und Jugendliche im psychiatrischen Kontext zu untersuchen. Sie wird des Weiteren eine der wenigen Forschungsvorhaben in Österreich darstellen, bei welcher das Stresserleben der ProbandInnen multidimensional erfasst wird. So wird die Veränderung des Stresserlebens sowohl hormonell mittels Messung des Cortisol-Levels als auch mittels subjektivem Selbstbericht untersucht. Als biologischer Indikator wird das Cortisol-Level im Speichel erfasst. Speichelcortisol stellt einen stressassoziierten Biomarker zur Erfassung der Aktivität der Hypothalamus-Hypophysen-Nebennierenrinden-Achse (HHNA) dar. Diese ist eines der zentralen biologischen Stress-Systeme des Körpers, die auf situative negative wie auch positive Umweltstimuli reagiert (Condon et al. 2018). Die Ergebnisse werden Erkenntnisse zur Wirkung der Clownbesuche auf psychologischer sowie hormoneller Ebene bieten und insofern auch einen Ausblick auf mögliche zukünftige klinische Forschungsfelder innerhalb gesundheitsfördernden Maßnahmen im kinder- und jugendpsychiatrischen Kontext liefern. Darüber hinaus wird die die Zielgruppe Behandlungspersonal im österreichischen Krankenhaussetting durch Befragungen zu den Effekten der Clownbesuche involviert.

Die Pilotstudie wird vor Studienbeginn als klinischer Trial präregistriert (voraussichtlich unter www.clinicaltrials.gov); ein Studienprotokoll wird zudem bei einer Fachzeitschrift eingereicht.

2.6. Beschreibung des Untersuchungsdesigns (z. B. Erhebungszeitpunkte, Kontrollgruppen, Anzahl der Gruppen, Fallzahlschätzung, Stichprobengewinnung u. dgl.)

In einem quasi-experimentellen Studiendesign ist eine Datenerhebung in zwei Phasen geplant. In einer **ersten Phase** werden in einem Zeitraum von vier aufeinanderfolgenden Wochen sowohl hormonelle als auch psychologische Stressindikatoren der Kinder und Jugendlichen erhoben. Im Zuge einer Pre-/Posttestung kommt es vor sowie nach dem Clownbesuch zur Entnahme einer Speichelprobe bei den ProbandInnen für die Messung des Cortisol-Levels. Psychologische Indikatoren des Stressempfindens werden zusätzlich anhand standardisierter Fragebögen erhoben. Zudem werden VertreterInnen des Personals der Institution in die Erhebung miteinbezogen. Mit Hilfe standardisierter Fragebögen wird die persönliche Wirkungswahrnehmung auf das Behandlungspersonal erhoben.

In einer **zweiten Phase** werden Kinder und Jugendliche nach drei Monaten erneut zu ihren Erinnerungen an die Clownbesuche befragt. Diese Befragung wird elektronisch erfolgen und es werden ausschließlich psychologische Befindensmaße mittels Fragebögen erhoben. Die zweite Phase zielt auf die Messung eines mittelfristigen Effekts der Clownbesuche ab.

Die Clownbesuche werden routinemäßig von BegegnungskünstlerInnen des Vereins ROTE NASEN Clowndoctors durchgeführt. Die Auswahl der Clowns, die Dauer des Besuchs sowie die spezifischen künstlerischen Abfolgen werden, einer herkömmlichen Situation gleich, nicht an das Forschungsvorhaben angepasst, sondern erfolgen nach intern organisierten Routineabläufen.

Zur Bestimmung der Stichprobengröße wurden frühere Referenzstudien herangezogen (Auerbach et al. 2016; Dionigi et al. 2013; Fernandez 2010; Hackl 2017; Leguizamon 2017; Lopes-Junior 2020; Rimon et al. 2016; Sanchez et al. 2017; Saliba et al. 2016; Zhang et al. 2017). Basierend auf den Effektgrößen früherer Referenzstudien wurden mit Hilfe der Software G*Power Mindestfallzahlen für die erwarteten Effekte berechnet. Effektgrößen des erwarteten Cortisol-Levels sowie der Befindlichkeitsmaße Stress, Angst, emotionales Befinden und Schmerzwahrnehmung liegen bei statistischen Annahmen (Power = 0.80, Alpha = 0.05) unter einer Gesamtanzahl von 30 Teilnehmenden. Auf Basis dieser Annahmen wird die Gesamtstichprobe mit mindestens 40 Kindern und Jugendlichen festgelegt.

Die ProbandInnen werden in psychiatrischen Einrichtungen angefragt. In Bezug auf die Auswahl der Kinder und Jugendlichen wird ein aufklärendes Erstgespräch mit dem behandelnden Personal geführt. Das Personal stellt darüber hinaus auch den Kontakt zu den Erziehungsberechtigten her, um die schriftliche Einverständniserklärung dieser einzuholen.

2.7. Beschreibung der Methode der Datenerhebung, der Stichprobe (StudienteilnehmerInnen), der Studienmaterialien (z. B. verwendete Instrumente) u. dgl.

Die Erhebung des Stresserlebens erfolgt in zwei Phasen in Kooperation mit klinischen Einrichtungen in verschiedenen Bundesländern Österreichs.

In der Vorbereitungsphase wird ein allgemeines Aufklärungsgespräch mit dem Behandlungsteam der kooperierenden Einrichtung geführt, um dieses über den genauen Ablauf der Studie sowie die angewendeten Erhebungsmethoden zu informieren. Das Behandlungsteam kann somit intern einen Ausschluss einzelner PatientInnen zum Schutz dieser entscheiden, ohne dem Studienteam spezifische Gründe darlegen zu müssen. Nachfolgend wird die schriftliche Einverständniserklärung der Erziehungsberechtigten in Kooperation mit dem Behandlungspersonal eingeholt. Aufgrund von Datenschutzbestimmungen sowie der Abwesenheit der Erziehungsberechtigen vor Ort wird von einer direkten Kontaktaufnahme durch das Projektteam abgesehen. Nachfolgend werden Kinder und Jugendliche individuell in einem Gespräch über die Studie informiert und im Beisein des Behandlungsteams um ihr Einverständnis gebeten.

Phase 1: Vor dem Eintreffen der Clowns werden die Kinder und Jugendlichen von der Versuchsleitung erneut über konkrete Details zum Ablauf der Erhebung aufgeklärt. Danach werden die ProbandInnen gebeten einen einmaligen Fragebogen zur Basisdatenerhebung auszufüllen, welcher rund 30 Minuten Zeit in Anspruch nimmt.
Im Anschluss kommt es zur Erhebung des aktuellen Befindens (Stress, Unruheempfinden, Stimmung) durch ein fünfminütiges Fragebogenset sowie der Entnahme einer Speichelprobe mit Hilfe einer Salivette. Hierbei werden die ProbandInnen aufgefordert, eine Baumwollrolle ca. ein bis zwei Minuten im Mund anzufeuchten. Sobald der Clownbesuch beendet ist, erfolgt eine weitere Speichelprobenentnahme. Das Fragebogenset zur Erhebung des aktuellen Befindens wird von den Kindern und Jugendlichen erneut ausgefüllt (alle verwendeten Erhebungsinstrumente sind dem Antrag beigefügt).

Das Studienpersonal steht den Kindern und Jugendlichen während der Datenerhebung davor und danach kontinuierlich für Fragen zur Verfügung, administriert die Speichelproben sowie koordiniert deren gesicherte Zwischenlagerung und Transport. Entnommene Proben werden in einem Kühlschrank der Einrichtung zwischengelagert bis sie nach Ende der letzten Datenerhebung wöchentlich vom Studienpersonal in die Räumlichkeiten des biochemischen Labors der Universität Wien transportiert und in einem abgesperrten Kühlschrank bei -20 Grad Celsius aufbewahrt werden.
Neben der Befragung der Kinder und Jugendlichen werden Personen des behandelnden Personals für eine schriftliche Befragung eingebunden. Die Befragung zielt auf die persönliche Wahrnehmung und Wirkungseinschätzung der Clownbesuche ab. Das Studienpersonal übernimmt hier erneut auch eine aufklärende Rolle, steht für Fragen zur Verfügung und bietet dem Personal Diskretion während des Ausfüllens des Fragebogens.
Genannte Abläufe der Phase 1 werden in wöchentlichen Abständen, am selben Wochentag in vier aufeinanderfolgenden Wochen wiederholt.

Phase 2: Nach drei Monaten werden selbige Kinder und Jugendliche für eine weitere Fragebogenerhebung angefragt. Mit Hilfe von Fragebogeninstrumenten werden erneut psychologische Marker des Stresserlebens und Erinnerungen an die Clownbesuche erhoben. Die ProbandInnen werden elektronisch informiert und gebeten, die Fragebögen elektronisch auszufüllen.

2.8. Geplanter Beginn und voraussichtliche Gesamtdauer der Studie
Hinweis: Bereits begonnene oder abgeschlossene Projekte werden von der Ethikkommission nicht begutachtet.

Die geplante Dauer der Studie ist mit maximal 18 Monaten angesetzt. Der Beginn des Forschungsvorhabens ist frühestmöglich mit Mai 2021 geplant. Die genaue Zeitplanung ist abhängig von der Begutachtung der Ethikkommission, dem Verlauf der SARS-CoV-2 Verbreitung sowie damit einhergehenden Einschränkungen von Clownbesuchen in den kooperierenden Einrichtungen.

3. StudienteilnehmerInnen

3a Rekrutierung der Teilnehmenden sowie Ein- und Ausschlusskriterien für die Studienteilnahme

3.1. Geplante Anzahl der Teilnehmenden

Geplant ist ein Einbezug von ProbandInnen von zwei klinischen, kinder- und jugendpsychiatrischen Einrichtungen mit je 20 Kindern und Jugendlichen sowie maximal fünf Personen des behandelnden Personals pro Einrichtung. Es ergibt sich somit eine totale Stichprobe von n = 40 Kindern und Jugendlichen und n = 10 Personen des Behandlungspersonals.

3.2. Voraussichtliche Zeitdauer der Studienteilnahme für die Teilnehmenden

Die Studienteilnehmenden werden in einem Zeitraum von vier aufeinanderfolgenden Wochen jeweils rund 20 Minuten an einer Pre-/Posttestung des Stresserlebens teilnehmen sowie an einer einmaligen Basisdatenerhebung, die 30 Minuten dauert. Nach einem Abstand von drei Monaten erfolgt eine erneute Datenerhebung ausschließlich psychologischer Indikatoren mittels Fragebogen. Hier wird ein Zeitaufwand von rund 20 Minuten berechnet. Insgesamt werden Kinder und Jugendliche demnach rund zwei Stunden in die Datenerhebung gebunden. Der gesamte Erhebungszeitraum erstreckt sich über vier Monate.

3.3. Charakterisierung der Teilnehmenden

- Mindestalter: 7 Höchstalter: 18
- Geschlecht: ☒ männlich ☒ weiblich
- Sind nicht persönlich Einwilligungsfähige einschließbar? ☐ Ja ☒ Nein
- Handelt es sich bei den Teilnehmenden um Kinder oder andere vulnerable Gruppen?

Ja. Die Zielgruppe der Studie sind Kinder und Jugendliche im Alter von 7 bis 18 Jahren, welche sich in einem stationären oder ambulanten psychiatrischen Setting befinden (mit unterschiedlichen Diagnosen).

3.4. Beschreiben Sie das geplante Rekrutierungsverfahren (bitte alle zur Verwendung bestimmten Materialien, z. B. Inserate, beilegen):

Die Auswahl der Kinder und Jugendlichen wird anhand mehrerer Schritte erfolgen. Der Verein ROTE NASEN Clowndoctors führt regelmäßige Clownbesuche in den folgenden Einrichtungen durch, die sich für eine Forschungskooperation bereit erklärten:
-) Tiroler Kliniken – Kinder und Jugendpsychiatrie Hall in Tirol
-) Klinik Hietzing – Kinder und Jugendpsychiatrie Rosenhügel
Aufbauend auf der bereits bestehenden Zusammenarbeit mit den genannten Institutionen wird der Verein ROTE NASEN Clowndoctors die logistische sowie administrative Unterstützung des Projekts übernehmen. Erste Kontakte in Bezug auf das wissenschaftliche Vorhaben wurden bereits mit Prim. Univ.-Prof.in Dr.in Kathrin Sevecke (Direktorin der Universitätsklinik für Psychiatrie, Psychotherapie und Psychosomatik im Kindes- und Jugendalter Innsbruck) sowie mit der OÄ Dr. Anna Kreuzeder (Interimistische Abteilungsleitung Kinder und Jugendpsychiatrie, Klinik Hietzing) aufgenommen, welche Ihre Bereitschaft zur Kooperation im genannten Projekt bestätigt haben.

Für eine erste Annäherung an die ProbandInnen ist die Kooperation mit dem Personal der Einrichtungen zentral. Bei einem vorherigen Aufklärungsgespräch mit dem behandelnden Team wird der Ablauf der Datenerhebung im Detail besprochen. Dem Behandlungsteam wird somit die Möglichkeit gegeben intern PatientInnen aus dem Forschungsvorhaben auszuschließen, sollte dies den Behandlungs- oder Genesungsverlauf dieser Kinder und Jugendlichen negativ beeinflussen oder aus anderen Gründen als nicht adäquat beurteilt werden.

In einem weiteren Schritt werden zuerst die Erziehungsberechtigten und nachfolgend die Kinder und Jugendlichen selbst um ihr Einverständnis zur Teilnahme angefragt.

3.5. Legen Sie kurz die Auswahl der Teilnehmenden sowie die Ein- und Ausschlusskriterien dar (wenn zutreffend: explizite Begründung für den Einschluss von Personen aus geschützten Gruppen, z. B. Minderjährigen, temporär oder permanent nicht einwilligungsfähigen Personen)

Minderjährige Kinder und Jugendliche stellen die primäre Zielgruppe von Clownbesuchen im psychiatrischen Kontext dar, weshalb die Fragestellung im Studienvorhaben auf diese Personengruppe abzielt. Die wissenschaftliche Relevanz des Themas ergibt sich auf Basis einer bestehenden Forschungslücke zur Wirkung von Clownbesuchen im psychiatrischen Kontext in Österreich. Bei der Auswahl der TeilnehmerInnen wird auf folgende Ein- und Ausschlusskriterien geachtet:

Kinder und Jugendliche
Einschlusskriterien: (a) Ambulante oder stationäre psychiatrische Behandlung in einer der kooperierenden Einrichtungen, (b) regelmäßige Teilnahme an Clownbesuchen, (c) Altersbereich 7 bis 18 Jahre, (d) Einverständnis der Erziehungsberechtigten und des Kindes/Jugendlichen.

Ausschlusskriterien: (a) fehlende Bereitschaft an Clownbesuchen teilzunehmen, (b) Einschätzung des behandelnden Personals zu negativen Folgen durch die Teilnahme an Clownbesuchen und/oder an der Datenerhebung.

Behandlungspersonal
Einschlusskriterien: (a) Behandlungsverantwortung (Medizinisches-, Paramedizinisches Personal, Bezugsbetreuungspersonal), (b) Kenntnisse des klinischen Diagnose- sowie Behandlungsbildes der Kinder und Jugendlichen.

Das Screening genannter Kriterien erfolgt durch qualifiziertes Studienpersonal im Gespräch mit dem behandelnden Personal, den Erziehungsberechtigten sowie den Kindern und Jugendlichen selbst.

3.6. Wird die Zustimmung der Teilnehmenden oder deren gesetzlicher Vertretung eingeholt?
☒ Ja (Informationsblätter und Einwilligungserklärung beilegen)
☐ Nein Wenn nein, warum nicht:

t.n.z.

3.7. Welche (persönlichen, sozialen, institutionellen) Beziehungen bestehen zwischen den Teilnehmenden und den Studiendurchführenden (z. B. Studierende-Lehrkraft, Dienstnehmer/in-Dienstgeber/in etc.)? Ist die Freiwilligkeit der Studienteilnahme gewährleistet?

Institutionelle Beziehungen bestehen zwischen dem Verein ROTE NASEN Clowndoctors sowie den kooperierenden Einrichtungen im psychiatrisch-klinischen Bereich. Der Verein ROTE NASEN Clowndoctors wird die logistische Unterstützung im Studienvorhaben übernehmen. Darüber hinaus wird darauf geachtet, dass die Begegnung im Zuge des Clownbesuchs möglichst unter Routinebedingungen stattfindet. Die Clowns bzw. andere MitarbeiterInnen des Vereins werden in keine Schritte der Datenerhebung oder -auswertung miteinbezogen.

Alle Teilnehmenden der Studie (Kinder, Jugendliche, behandelndes Personal) werden vorab mündlich über den Studienverlauf, mögliche Folgen, die Freiwilligkeit der Teilnahme sowie den jederzeit möglichen Abbruch ohne Anführung von Gründen aufgeklärt. Dieser Informationsprozess wird sowohl mit den Erziehungsberechtigten als auch mit den Kindern und Jugendlichen selbst in altersgerechter Form durchgeführt. Besonderes Augenmerk wird auf die Rechte und den Schutz der Minderjährigen gelegt, worauf auch das erste Aufklärungsgespräch mit dem behandelnden Personal abzielt.

3b Datenschutz

Bitte unterscheiden Sie Anonymisierung und Pseudonymisierung!
Anonymisierung: Daten können einer Person unwiderruflich nicht oder nicht mehr zugeordnet werden. Daher unterliegen anonyme und anonymisierte Daten keinem Datenschutz.
Pseudonymisierung: Daten lassen sich einer Person mit zusätzlichen Informationen (meist einem Identifikationsschlüssel) zuordnen (Art. 4 Z 5 DSGVO) und bleiben daher personenbezogene Daten. Als solche unterliegen sie dem Datenschutz.

3.8. Welche personenbezogenen (d.h. Informationen, die sich auf eine identifizierte oder identifizierbare natürliche Person beziehen) Daten werden erhoben?

Vor der gesundheitsspezifischen Datenerhebung in Phase 1 werden folgende personenbezogene Daten der Kinder und Jugendlichen abgefragt: Name, Geschlecht, Geburtsdatum, Muttersprache, Email Adresse.

Zur Erhebung der Einschätzung des Personals erfolgt ebenfalls eine Dokumentation personenbezogener Daten: Name, Geschlecht, Alter, Position, Zeit in der Position in der kooperierenden Einrichtung, Arbeitsstundenausmaß.

Im Rahmen der Erhebung psychologischer Stressmaße werden darüber hinaus gesundheitsspezifische Daten festgehalten. Darunter fallen die Einschätzung des persönlichen Wohlbefindens sowie Trait- und State-Messdaten. Es kommen dafür standardisierte Fragebögen des Fachbereichs Klinische Psychologie zur Anwendung, welche als unbedenklich eingestuft werden.

3.9. Wie wird die Anonymität der Teilnehmenden gewährleistet?

Die Studie unterliegt den aktuellen Bestimmungen der europäischen Datenschutzverordnung (DSGVO) sowie den jeweiligen einrichtungsspezifischen Richtlinien zum Umgang mit medizinischen Daten. Alle personenbezogenen sowie gesundheitlichen Daten, die im Verlauf der Studie erhoben werden, werden anhand einer Codevergabe pseudonymisiert gespeichert. Das Studienpersonal, welche die Datenerhebung vor Ort koordiniert, stellt eine vertrauliche Behandlung der Daten sowie eine zeitnahe Überlieferung der Daten an die Projektleitung sicher. Die Speicherung aller Daten erfolgt auf einem geschützten Datenserver der Universität Wien mit ausschließlicher Zugriffsbefugnis durch die Projektleitung. Ein nachträglicher Rückschluss individualisierter Daten ist ausschließlich im Zuge eines Studienaustritts von ProbandInnen zum Zweck der Datenvernichtung oder auf Einsichtswunsch der Teilnehmenden bzw. der Erziehungsberechtigten möglich.
Nach Abschluss der Studie werden alle Daten vollständig anonymisiert und unbefristet auf einem geschützten Datenserver der Universität Wien aufbewahrt. Kontaktdaten werden zu diesem Zeitpunkt unwiderruflich gelöscht.

3.10. Wenn eine vollständige Anonymisierung personenbezogener Daten nicht möglich ist: Wie wird die Privatsphäre der Teilnehmenden geschützt?

t.n.z.

3.11. Werden Stimmen, Bilder oder Videos aufgenommen?

☐ Ja ☒ Nein

Wenn Ja: Wird die Einwilligung der Teilnehmenden zur Aufnahme eingeholt?

t.n.z.

3.12. Wie wird gewährleistet, dass die Teilnehmenden jederzeit die laufende Mitwirkung an der Studie abbrechen können?

Vor Beginn der Datenerhebung werden alle Studienteilnehmenden (Kinder, Jugendliche, behandelndes Personal) sowie im Falle von Minderjährigkeit deren Erziehungsberechtige, über den gesamten Studienverlauf, die Freiwilligkeit der Studienteilnahme sowie über das jederzeitige Abbruchsrecht ohne Angabe von Gründen in mündlicher und schriftlicher Form informiert. Es wird darüber hinaus darauf hingewiesen, dass für die ProbandInnen keinerlei Nachteile oder Konsequenzen im Falle eines Studienabbruchs entstehen werden.

3.13. Wie erfolgt die Verarbeitung und Auswertung der Studiendaten?

☐ personenbezogen, Begründung:

t.n.z

☒ indirekt personenbezogen Wie erfolgt die Anonymisierung?

Alle erhobenen Daten werden in einem ersten Schritt pseudonymisiert. Hierfür erfolgt eine Codevergabe, anhand welcher das Matching der mehrfach erhobenen Daten derselben ProbandInnen ermöglicht wird, jedenfalls aber keine Personifizierung zulässt. Der Zugriff auf die Codierung ist ausschließlich der Projektleitung anhand einer Codierungsliste möglich. Diese wird auf einem geschützten Datenserver der Universität Wien gespeichert.<br>Ein frühzeitiger Abbruch der Studienteilnahme sowie der Wunsch eines Teilnehmenden oder deren Erziehungsberechtigten zur Einsicht sind ausschließliche Gründe, im Zuge welcher eine Entschlüsselung möglich gemacht wird.<br>Einen Monat nach Abschluss der Studie erfolgt eine vollständige Anonymisierung aller Daten, wodurch eine Personifizierung der Daten ausgeschlossen wird.

Hinweis: Bitte beachten Sie, dass es aus datenschutzrechtlicher Sicht empfehlenswert ist, ausschließlich anonymisierte Daten zu verwenden.

3.14. Wo, wie lange und in welcher Form werden die folgenden Daten aufbewahrt bzw. gespeichert? Für welche Personen sind diese Daten zugänglich?

a. Einverständniserklärung

Schriftliche Einverständniserklärungen von allen Teilnehmenden (Kinder, Jugendliche, Erziehungsberechtigte, behandeltes Personal) werden vom Studienpersonal zeitnah an die Projektleitung weitergeleitet. Diese hat ausschließlichen Zugang zu den unterzeichneten Erklärungen welche in gesicherten Räumlichkeiten der Universität Wien unbefristet abgelegt werden.

b. Studiendaten (Daten, welche im Laufe der konkreten Studie erhoben werden. Z. B. Mess-Rohdaten, Audio- und Videomaterial, Beobachtungsprotokolle, Transkripte von Interviews und Fokusgruppen etc.)

Ausgefüllte pseudonymisierte Fragebögen werden zeitnah vom Studienpersonal an die Projektleitung übergeben. Diese stellt die Ablegung der papier-basierten Daten in gesicherten Räumlichkeiten der Universität Wien sicher. Ein Monat nach Abschluss der Studie werden die Daten unwiderruflich anonymisiert und eine Einsicht zur Personifizierung ist nicht mehr möglich.

c. Anamnesebögen

t.n.z.

d. Humanproben (z. B. Blutproben, Speichelproben)

Die verwendeten Salivetten mit den erhobenen Speichelproben werden in der klinischen Einrichtung, in der die Erhebung durchgeführt wird, in institutseigenen Kühlschränken zwischengelagert. Die Beschriftung erfolgt bereits pseudonymisiert. Nach Abschluss der Erhebung vor Ort werden die Proben vom Studienpersonal wöchentlich zeitnah in das Analyselabor transportiert.

3.15. Wenn Studiendaten *nicht* in anonymisierter bzw. pseudonymisierter Form aufbewahrt werden: Wo und warum ist dies der Fall?

t.n.z.

3.16. Welche Sicherungsmaßnahmen werden ergriffen, um die Daten der Teilnehmenden vor unberechtigtem Zugriff zu schützen? (z. B. Virenschutz/Firewall, Individueller Log-In und Kennwortverfahren, Verwaltung

von Berechtigungen, Verschlüsselung von Systemen, elektronisches Zutrittskontrollsystem, Sicherheitstüren).

Eine Pseudonymisierung aller erhobener Daten (Fragebögen, Salivetten mit Speichelproben) zeitnah nach der Erhebung ermöglicht den Datenschutz und verhindert den Zugriff durch unerwünschte Dritte.

Salivetten werden zum Zeitpunkt der Erhebung in Phase 1 in den klinischen, institutionseigenen Kühlschränken zwischengelagert, zu welchen ausschließlich das Personal der Einrichtung sowie das Studienpersonal Zugang hat. Das Personal wird über die logistischen Prozesse ausführlich informiert und ist darüber hinaus zu Vertraulichkeit im Umgang mit medizinischen Daten verpflichtet.

Alle erhobenen Daten werden in vollständig anonymisierter Form auf einem geschützten Datenserver der Universität Wien unbefristet und unzugänglich für Unbefugte aufbewahrt. Die Datenserver der Universität Wien sind passwortgeschützt und sind nur nach erfolgter Autorisierung durch den Zentralen Informatikdienst der Universität Wien zugänglich. Es können ausschließlich autorisierte und zur Verschwiegenheit verpflichtete Beauftragte von in- und/oder ausländischen Gesundheitsbehörden und jeweils zuständige Ethikkommissionen in die nicht-verschlüsselten Daten Einsicht nehmen, soweit dies für die Überprüfung der ordnungsgemäßen Durchführung der Studie notwendig bzw. vorgeschrieben ist. Sämtliche Personen, die Zugang zu den verschlüsselten und nicht-verschlüsselten Daten erhalten, unterliegen im Umgang mit den Daten der DSGVO sowie den österreichischen Anpassungsvorschriften in der jeweils gültigen Fassung. Ebenfalls bestätigt die Versuchsleitung mit ihrer Unterschrift, dass sie sämtliche Angaben sowie persönliche Informationen von teilnehmenden Personen, die sie während ihrer Arbeit in der Studie erfahren, streng vertraulich behandeln.

3.17. Können sich Teilnehmende über die Forschungsergebnisse informieren?

☒ Ja ☐ Nein Wenn nein, Begründung:

Die Teilnehmenden haben während der Studienphase die Möglichkeit, sich bei etwaigen Fragen an die in der Einverständniserklärung angegebenen Kontaktpersonen zu wenden. Darüber hinaus werden die Ergebnisse den kooperierenden Institutionen in schriftlicher und anonymisierter Form zur Verfügung gestellt. Auch die Studienteilnehmenden haben die Möglichkeit, den Endbericht einzusehen. Hierfür ist ein formloses schriftliches Interesse gegenüber der Projektleitung auszudrücken.

3.18. Wie wird den Teilnehmenden die Einsicht in ihre persönlichen Studiendaten ermöglicht?

Eine Einsicht in die erhobenen pseudonymisierten Daten wird den Teilnehmenden oder ihren gesetzlichen Vertretungen auf Nachfrage hin bis einen Monat nach der Beendigung der Studie ermöglicht. Hierfür ist ein schriftlicher, formloser Antrag an die Projektleitung zu stellen, welche nachfolgend einen Termin zur Einsicht vereinbaren wird. Die Einsicht erfolgt in den Örtlichkeiten der Universität Wien.

3.19. Auf welche Weise und wie lange können die Teilnehmenden die Löschung ihrer Studiendaten verlangen?

Eine nachträgliche Löschung der erhobenen pseudonymisierten Daten kann von den Teilnehmenden bis einen Monat nach Beendigung der Studie beantragt werden. Hierfür ist ein formloser Antrag an die Projektleitung schriftlich zu verfassen. Dieser Antrag wird ebenso schriftlich von der Projektleitung freigegeben, welche auch die Löschung durchführt.

3c Folgen der Studienteilnahme für die Teilnehmenden

3.20. Ist die Teilnahme an der Studie für die Teilnehmenden mit absehbaren Risiken oder anderen anzunehmenden problematischen Begleiterscheinungen verbunden (z. B.: Schmerzen, Unannehmlichkeiten oder Verletzungen der persönlichen Integrität)? Welche Maßnahmen werden zur Vermeidung und/oder Versorgung von unvorhergesehenen/unerwünschten Ereignissen getroffen?

In Bezug auf die Studienabläufe sind keine Gefährdungen der psychischen oder physischen Integrität der Teilnehmenden im Sinne negativer, schädigender Outcomes zu erwarten. Jegliche Form der Datenerhebung in der geplanten Studie ist als unbedenklich für die Studienteilnehmenden einzustufen, inklusive der Entnahme von Speichelproben. Die Messungen sind ungefährlich, non-invasiv und mit keinerlei Schmerzen oder bekannten Risiken verbunden. Alle verwendeten Geräte sind für Untersuchungen an Minderjährigen zugelassen. Darüber hinaus werden die teilnehmenden Kinder vor Beginn der Testungen über die Apparatur und die Art des Messverfahrens kindgerecht informiert.

Bei der Beantwortung der Fragebögen ist es unter Umständen möglich, dass bei den Teilnehmenden unerwartete oder unangenehme Gefühle aufkommen. Die ProbandInnen werden vorab darüber aufgeklärt, dass sie in einem solchen Fall den Kontakt zur Versuchsleitung oder zum behandelten Personal aufsuchen sollten.

Ein besonderer Fokus wird auf ein ausführliches Aufklärungsgespräch mit dem medizinischen Behandlungspersonal sowie auf eine altersgerechte Aufklärung der ProbandInnen gelegt. Dadurch können das Risiko negativer Konsequenzen für den Behandlungsverlauf durch die Studienteilnahme sowie das Risiko einer unfreiwilligen Teilnahme der Kinder und Jugendlichen ausgeschlossen werden.

3.21. Welche Verfahren werden eingesetzt, um unerwünschte Effekte einer Studienteilnahme zu identifizieren, diese zu dokumentieren und zu berichten? Beschreiben Sie, wann, durch wen und wie dies erfolgt, z. B. freies Befragen und/oder an Hand von Fragelisten.

In einem ersten Aufklärungsgespräch mit dem behandelnden Personal der Kinder und Jugendlichen wird diesem die Möglichkeit gegeben, eine Teilnahme an der Studie in Hinblick auf mögliche Risiken für den klinischen Behandlungsverlauf jedes Kindes bzw. Jugendlichen intern abzuklären. Es wird auf eine ausführliche Aufklärung jeglicher Prozesse sowie eine Einsicht in die Erhebungsinstrumente der Studie Wert gelegt. Entscheidungen für den Ausschluss eines Kindes oder Jugendlichen durch das behandelnde Personal müssen keinesfalls gegenüber dem Projektteam gerechtfertigt werden und werden in Ausschluss der Projektverantwortlichen erfolgen.

Im Informationsgespräch mit den Kindern und Jugendlichen wird besonderer Wert auf die Aufklärung bezüglich der Rechte der TeilnehmerInnen in altersgerechter Form gelegt. Hervorgehoben in diesem Gespräch sowie der schriftlichen Ausführung werden im Speziellen die Aspekte Freiwilligkeit und jederzeitiges Abbruchsrecht der Teilnahme sowie die Möglichkeit jederzeitiger Kontaktaufnahme mit der Projektleitung im Falle von Fragen oder Anliegen.

Die Datenerhebung selbst (Fragebögen, Entnahmen der Speichelprobe) erfolgt diskret, aber in Anwesenheit vom Studienpersonal sowie im Falle der Speichelentnahme auch einer Person des Behandlungsteams. Die Teilnehmenden erhalten so die unmittelbare Möglichkeit der Kontaktaufnahme zur Klärung im Falle von auftretenden Schwierigkeiten.

3.22. Wird eine Teilnahmevergütung bzw. eine Aufwandsentschädigung (Ausgleich von Fahrtspesen und Einkommensentgang) an die Teilnehmenden bezahlt?

Die Teilnahme an der Studie ist für die ProbandInnen mit keinerlei Kosten verbunden. Die Datenerhebung wird in den Räumlichkeiten der kooperierenden klinischen Einrichtungen (Phase 1) oder elektronisch (Phase 2) erfolgen. Erziehungsberechtigte werden telefonisch oder elektronisch kontaktiert.

Die Teilnahme wird mit geringer zeitlicher Belastung kalkuliert. Eine monetäre Vergütung der Teilnahme ist nicht vorgesehen. Als kleine Aufwandsentschädigung stellt der Verein ROTE NASEN Clowndoctors allen Teilnehmenden eine Überraschungsbox zur Verfügung. Zusätzlich wird den teilnehmenden Kindern und Jugendlichen eine Urkunde zur Teilnahme am Forschungsprojekt ausgestellt.

3.23. Welche voraussichtlichen Vorteile bzw. welcher mögliche Nutzen sind für die Teilnehmenden mit der Studie verbunden?

Die teilnehmenden Kinder und Jugendlichen erhalten Einblick in den Ablauf einer quasi-experimentellen Studie im Bereich der Stressforschung. Nach Abschluss der Studie wird allen Teilnehmenden Zugang zum anonymisierten Abschlussbericht über die Resultate ermöglicht, worauf sie individuell schriftlich hingewiesen werden. Auf eine altersgerechte Vermittlung wird geachtet.
Das Personal der kooperierenden Einrichtungen hat die Möglichkeit, einen Beitrag zur wissenschaftlichen Bearbeitung einer Fragestellung innerhalb ihres Arbeitsfeldes zu leisten. Die erzielten Ergebnisse haben darüber hinaus praktischen Nutzen für teilnehmendes Personal der Einrichtungen. Die Wissenserweiterung kann einen Beitrag zur professionellen Versorgung der Zielgruppe leisten.

3d Weitere ethische Aspekte

3.24. Werden die Teilnehmenden in vollem Umfang über Art, Ziel und Inhalt der Studie informiert?

☒ Ja ☐ Nein, Begründung:

t.n.z.

Werden die Teilnehmenden getäuscht?

☐ Ja ☒ Nein

Wenn ja: Beschreibung und Begründung der Notwendigkeit der Täuschung:

t.n.z.

3.25. Welche weiteren möglicherweise auftretenden Probleme bzgl. Studienteilnahme und Studiendurchführung gibt es aus Ihrer Sicht?

Nach Einschätzung der Projektleitung kann es zu keinen gravierenden Problemen hinsichtlich der Studienteilnahme bzw. -durchführung kommen.

3.26. In welchem Verhältnis stehen potenzielle Risiken der Studie zum erwarteten wissenschaftlichen und gesellschaftlichen Nutzen?

Potentielle Risiken der Studie werden als sehr gering gewertet. Die wissenschaftliche Relevanz der Thematik sowie der gesellschaftliche und gesundheitsrelevante Nutzen der Studie überwiegen unserer Ansicht nach die potentiellen Risiken.

3.27. Unter welchen Bedingungen ist eine Unterbrechung der Studie vorgesehen? Unter welchen Umständen wird die Studie gänzlich abgebrochen?

Die Projektleitung erkennt keinen Grund, dass die Studie gänzlich vorzeitig abgebrochen werden muss. Aufgrund der aktuellen globalen Restriktionen im Zuge der SARS-CoV-2 Verbreitung könnte es zu einem Aufschub der geplanten Datenerhebung kommen, sollten Clownbesuche in den kooperierenden Einrichtungen aufgrund von Maßnahmen zur Infektionsverbreitung eingeschränkt werden.

Die Projektleitung hält sich dennoch vor, die Teilnahme von gewissen Personen während des Studienverlaufs zu beenden. Gründe hierfür können sein, dass die Person merklich kein Interesse an der Erhebung zeigt bzw. den Anforderungen der Erhebungsinstrumente nicht entsprechen kann oder ihr Gesundheitszustand durch die Teilnahme beeinträchtigt wird. Diese Bedingung ist im Informationsblatt für die TeilnehmerInnen schriftlich festgehalten.

4. Sonstige Anmerkungen

keine

5. Studiendurchführende

Geben Sie alle an der Studie Mitarbeitenden an:

Bitte berücksichtigen Sie dabei ggf. die Abgrenzung der Funktionen Projektleitung (Leitung des Gesamtprojekts), Studienleitung (Leitung einer Teilstudie) und Versuchsleitung (Durchführung einer Studie). Diese unterschiedlichen Funktionen bzw. Zuständigkeiten sollen auch in der TeilnehmerInneninformation und Einverständniserklärung entsprechend abgebildet werden.

Name	Institution	Funktion*	Qualifikation**
Univ.-Prof. Dr. Martina Zemp	Institut für Klinische und Gesundheits-psychologie, Fakultät für Psychologie, Universität Wien	Hauptprojektleitern (PI)	Universitätsprofessorin, Psychotherapeutin
Prof. Dr. Urs Nater	Institut für Klinische und Gesundheits-psychologie, Fakultät für Psychologie, Universität Wien	Projektleiter (Co-PI)	Universitätsprofessor
MMag.a Simone Seebacher MA	ROTE NASEN Clowndoctors	Projektkoordinatorin	MMag., MA
Amos Friedrich, BSc	Institut für Klinische und Gesundheits-psychologie, Fakultät für Psychologie, Universität Wien	Studiendurchführung/ Versuchsleitung	M.Sc. cand.
Lorena Holzmeier, BSc	Institut für Klinische und Gesundheits-psychologie, Fakultät	Studiendurchführung/ Versuchsleitung	M.Sc. cand.

	für Psychologie, Universität Wien		

* z. B. Studienleitung, Projektleitung, Versuchsleitung, Planung, Auswertung, Datenerhebung

** z. B. Senior Researcher, Dissertant/in, Postdoc usw.

Gibt es Interessenskonflikte zwischen den beteiligten ForscherInnen?

☐ Ja ☒ Nein

Wenn Ja: Beiblatt Interessenskonflikt ausfüllen und beilegen.

6. Name und Unterschrift der antragstellenden Person

Name: Prof. Dr. Martina Zemp
Institution/Firma: Arbeitsbereich Klinische Psychologie des Kindes- und Jugendalters, Fakultät für Psychologie, Universität Wien
Position: Universitätsprofessorin

Unterschrift der antragstellenden Person: Hiermit bestätige ich, dass die in diesem Antrag gemachten Angaben zur geplanten Studie korrekt sind und die Studie gemäß diesen Angaben und in Übereinstimmung mit den Prinzipien guten wissenschaftlichen Arbeitens durchgeführt wird.

Ich versichere, den empirischen Projektteil der eingereichten Studie (Rekrutierung, Datenerhebung) nicht vor dem Votum der Ethikkommission zu beginnen.

Wien, 22.3.2021

Unterschrift der Antragstellerin/des Antragstellers, Datum

6.1. Zustellungsbevollmächtigte/r falls nicht antragstellende Person

Application form for the assessment of planned academic studies by the Ethics Committee of the University of Vienna

Please enclose the study proposed. In addition (!) to your brief answers (no more than 150 words) to the following questions, you can refer to the relevant section in the application form.

Please use not applicable (n/a) for points that do not apply.

The Ethics Committee points out that it will only process applications that fulfil all formal requirements. If additions are necessary due to formal reasons or if certain information has not been provided (e.g. questionnaires), the application will be processed at a later meeting.

1. General information

1.1. Name of the applicant

Univ.-Prof. Dr. Martina Zemp

1.2. Title of the study

Effects of clown visits on stress and mood in children and adolescents in a psychiatric context

1.3. The study submitted is:

☐ a third-party-funded project

☐ proposal submitted ☐ already approved

Funding body/internal university project number/cost centre (if available):

☒ an internal university project

☐ a doctoral thesis (PhD thesis)

Name of the supervisor:

The supervisor was notified about the submission

☐ Yes ☐ No

Date of the successful completion of the public presentation at the faculty

☐ a master's thesis/diploma thesis

Name of the supervisor:

Application for submission made by:

☐ Supervisor ☐ Body responsible for study matters

1.4. Why and/or for what purpose do you need a vote of the Ethics Committee?

☐ The study could threaten the research subject's physical and psychological integrity, the right to privacy, other subjective rights or other prevailing interests.

☐ A publication medium demands the vote of the Ethics Committee.
☐ A funding body demands the vote of the Ethics Committee.
☒ Other reasons, namely:

The research project is carried out in cooperation with the association ROTE NASEN Clowndoctors and clinical institutions of the health sector (e.g. child and adolescent psychiatry). The evaluation by an ethics committee is required by the institutions as well as for the protection of the study participants. The research project will also be submitted for ethical review at the respective state level if requested by the collaborating institutions.

1.5. Has an ethics committee already evaluated this study?

☐ Yes ☒ No If yes, please enclose the relevant report.

2. Brief information about the planned study

2.1. Is the planned study part of a larger research project? If yes: Please specify the title of this larger research project.

n.a.

2.2. Academic discipline

Psychology, Clinical Psychology of Childhood and Adolescence

2.3. Brief description of the planned study (no more than 500 words)

The study project is a pilot study to measure short- and medium-term effects of clown visits of the association ROTE NASEN Clowndoctors. The focus is on the stress experience of children and adolescents in inpatient or outpatient psychiatric contexts who experience clown visits in the institutions at weekly intervals. In a quasi-experimental study design, the stress experience of the subjects is assessed by means of hormonal markers and questionnaires using a pretest/posttest. The target sample involves 40 children and adolescents who are treated as outpatients or inpatients in psychiatric institutions in Austria and who participate in clown visits of the association ROTE NASEN Clowndoctors at weekly intervals.

A pretest/posttest measurement of the cortisol level before and after the clown visit will take place in four consecutive weeks. With the help of standardized questionnaires, additional psychological stress measures (subjective stress experience, current feeling of restlessness as well as positively perceived affect by the clown visit) will be recorded in the participants. The application of standardized questionnaires in this phase is paper-based (paper-pencil) and takes place in the presence of the experimental supervisor. The measurement of the cortisol level is done using saliva samples. The saliva swab of the children and adolescents will be taken with the help of a salivette once before and once after the interaction with the clowns. For this purpose, the subject will be asked to moisten a cotton roll in the oral cavity for one to two minutes. All collected data will be treated pseudonymously after the field survey.
The clown visit is organized and carried out by encounter artists of the association ROTE NASEN Clowndoctors in a conventional way. Furthermore, these artists are not included in the data collection.

The study is conducted under the direction of the Department of Clinical and Health Psychology of the Faculty of Psychology (PI: Prof. Dr. Martina Zemp; Co-PI: Prof. Dr. Urs Nater) in cooperation with the association ROTE NASEN Clowndoctors (Coordinator: MMag. Simone Seebacher MA).

2.4. Objectives of the study (academic questions, hypotheses, etc.)

The aim of the research project is to investigate the short- and medium-term effects of clown visits on the hormonal and psychological stress experience of children and adolescents in a psychiatric context. A stress-reducing effect of clown visits on the target group is expected, which will be measured by biopsychological markers. Specifically, the following hypotheses will be tested:

1. Children and adolescents show reduced cortisol levels as well as a lower subjective stress experience after the clown visit (post measurement) compared to before (pre-test).
2. The more frequently the children and adolescents participated in the clown visits within the four-week data collection period, the stronger the stress-reducing effects.
3. The subjective assessment of the treatment staff indicates positive effects of the clown visits on the mood of the treatment staff, the atmosphere in the hospital ward and the well-being of the children and adolescents.

2.5. Academic and social relevance of the study

Experiences of illness and hospitalization are often associated with stress, anxiety, and tension for patients and their families. Experiences of pain, loss of control, confusion, and separation from close family members can lead to physical as well as mental health impairments (Price et al. 2016). Symptoms of negative stress have also been identified in children following discharge from a hospital context, including increased agitation as well as decreased self-esteem (Rennick & Rashotte 2009).

Clown visits have been taking place for decades in health care facilities, where humor, distraction and creativity can be used to reinterpret the stressful situation and thus provide relief for those affected. International studies have already pointed out the effectiveness of clown visits in relation to different outcomes. It has been shown that interaction with clowns prior to an invasive medical procedure can reduce perceived stress and tension (Vagnoli et al. 2005; Dionigi et al. 2014). A positive effect of clown attendance has also been studied in relation to the pain experience of children undergoing particularly painful examinations (Goldberg et al. 2014; Ben-Pazi et al. 2017). In addition, results show a tension-reducing effect comparable to sedation (Viaggiano et al. 2015).
Isolated studies have also uncovered effects of clown visits on treatment staff and daily work in the hospital setting. The presence of clowns is appreciated by anesthesiologists especially before and after surgery (Smerling et al. 1999). A very positive effect was found within the nursing staff on children's wards, where changes have been observed in both communication with patients and the general daily work routine (Blain et al. 2012).

The health-promoting effect of clown visits has already been studied from different angles. However, the effect on specific target groups as well as the collection of biopsychological markers has not been sufficiently researched to date. The present pilot study will contribute to investigate the effect of clown visits on children and adolescents in a psychiatric context. Furthermore, it will be one of the few research projects in Austria in which the stress experience of participants is recorded multidimensionally. Thus, the change in stress experience will be investigated both hormonally by measuring cortisol levels and by subjective self-report. As a biological indicator, the cortisol level in saliva is recorded. Salivary cortisol represents a stress-associated biomarker for detecting the activity of the hypothalamic-pituitary-adrenocortical axis (HHNA). This is one of the body's central biological stress systems that responds to situational negative as well as positive environmental stimuli (Condon et al. 2018). The results will offer

insights into the effect of clown visits on psychological as well as hormonal levels and in this respect also provide a perspective on possible future clinical research fields within health promoting interventions in the child and adolescent psychiatric context. Furthermore, the target group of treatment staff in the Austrian hospital setting will be involved through surveys on the effects of the clown visits.

The pilot study will be preregistered as a clinical trial prior to study initiation (anticipated at www.clinicaltrials.gov); a study protocol will also be submitted to a peer-reviewed journal.

2.6. Description of the research design (e.g. survey dates, control groups, number of groups, estimated sample size, sampling, etc.)

Both hormonal and psychological stress indicators of the children and adolescents will be collected over a period of four consecutive weeks. In a pre/posttest design, a saliva sample will be taken from the participants before and after the clown visit to measure the cortisol level. Psychological indicators of the stress perception are additionally collected by means of standardized questionnaires. Furthermore, representatives of the institution's staff will be included in the survey. Standardized questionnaires will be used to collect personal perceptions of impact on treatment staff.

The clown visits are routinely carried out by encounter artists of the association ROTE NASEN Clowndoctors. The selection of the clowns, the duration of the visit as well as the specific artistic sequences are, similar to a conventional situation, not adapted to the research project, but take place according to internally organized routines.

Previous reference studies were used to determine sample size (Auerbach et al. 2016; Dionigi et al. 2013; Fernandez 2010; Hackl 2017; Leguizamon 2017; Lopes-Junior 2020; Rimon et al. 2016; Sanchez et al. 2017; Saliba et al. 2016; Zhang et al. 2017). Based on effect sizes from previous reference studies, minimum case sizes for expected effects were calculated using G*Power software. Effect sizes of expected cortisol level and measures of well-being stress, anxiety, emotional well-being, and pain perception are below a total number of 30 participants with statistical assumptions (power = 0.80, alpha = 0.05). Based on these assumptions, the total sample is determined to be at least 40 children and adolescents.

The participants are approached about the study in the psychiatric institutions. With regard to the selection of children and adolescents, an informative initial interview is conducted with the treating staff. The staff also establishes contact with the legal guardians in order to obtain their written consent.

2.7. Description of the data collection method, the sample (participants in the study), materials used for the study (e.g. instruments used), etc.

The survey of experienced stress is conducted in two phases in cooperation with clinical institutions in different provinces of Austria.

In the preparatory phase, a general educational discussion is held with the treatment team of the cooperating institution to inform them about the exact procedure of the study and the data collection methods used. The treatment team can thus decide internally to exclude individual patients for their protection without having to explain specific reasons to the study team. Subsequently, the written informed consent of the legal guardians is obtained in cooperation with the treatment staff. Due to data protection regulations and the absence of the legal guardians on site, the project team will not contact

them directly. Subsequently, children and adolescents are informed individually about the study in a conversation and asked for their consent in the presence of the treatment team.

Before the clowns arrive, the children and adolescents are again informed by the test administration about specific details of the survey procedure. Afterwards, the participants are asked to fill out a one-time questionnaire for basic data collection, which takes about 30 minutes.
Subsequently, the current state of health (stress, restlessness, mood) is assessed by means of a five-minute questionnaire set and the collection of a saliva sample with the aid of a salivette. Here, the subjects are asked to moisten a cotton roll in their mouth for about one to two minutes. As soon as the clown visit is over, another saliva sample is taken. The questionnaire set for surveying the current state of health is filled out again by the children and adolescents (all survey instruments used are attached to the application).
The study staff is continuously available to the children and adolescents for questions during the data collection before and after, administers the saliva samples and coordinates their secure interim storage and transport. Collected samples are temporarily stored in a refrigerator at the facility until they are transported weekly by study staff to the premises of the biochemical laboratory of the University of Vienna after the end of the last data collection and stored in a locked refrigerator at -20 degrees Celsius.
In addition to the survey of the children and adolescents, persons of the treating staff will be involved for a written survey. The survey aims at the personal perception and impact assessment of the clown visits. Here, the study staff again also assumes an informative role, is available for questions and offers the staff discretion during the completion of the questionnaire.
Mentioned procedures of phase 1 are repeated at weekly intervals, on the same day of the week for four consecutive weeks.

2.8. Planned start and expected duration of the study
Please note: The Ethics Committee does not evaluate projects that have already started or that have already been completed.

The planned duration of the study is a maximum of 18 months. The start of the research project is planned for May 2021 at the earliest. The exact timing depends on the review of the ethics committee, the course of the SARS-CoV-2 spread and the associated restrictions on clown visits in the cooperating institutions.

3. Participants in the study

3a Recruitment of participants and inclusion and exclusion criteria for participating in the study

3.1. Planned number of participants

It is planned to include subjects from two clinical, child and adolescent psychiatric facilities, each with 20 children and adolescents and a maximum of five persons of the treating staff per facility. This results in a total sample of n = 40 children and adolescents and n = 10 persons of the treatment staff.

3.2. Expected duration of participation in the study

Over a period of four consecutive weeks, the study participants will each take part in a pre/posttest of stress experience for around 20 minutes, as well as a one-off baseline data collection session lasting 30 minutes. In total, children and adolescents are therefore involved in the data collection for around two hours. The entire data collection period extends over five weeks.

3.3. Characterisation of participants

• Minimum age: 7 Maximum age: 18
• Gender: ☒ male ☒ female
• Does the study include persons who are not able to give their consent personally? ☐ Yes ☒ No
• Are the participants children or other vulnerable persons?

Yes. The target population of the study is children and adolescents aged 7 to 18 years who are in an inpatient or outpatient psychiatric setting (with various diagnoses).

3.4. Please outline the planned recruitment procedure (attach all the material used for that purpose, such as advertisements):

The selection of children and adolescents will be based on several steps. The association ROTE NASEN Clowndoctors conducts regular clown visits in the following institutions that agreed to cooperate in research:
-) Tyrolean Clinics - Child and Adolescent Psychiatry Hall in Tyrol
-) Hietzing Clinic - Child and Adolescent Psychiatry Rosenhügel
Based on the already existing cooperation with the mentioned institutions, the association ROTE NASEN Clowndoctors will take over the logistic as well as administrative support of the project. First contacts regarding the scientific project have already been made with Prim. Univ.-Prof.in Dr.in Kathrin Sevecke (Director of the University Clinic for Psychiatry, Psychotherapy and Psychosomatics in Childhood and Adolescence Innsbruck) as well as with OÄ Dr. Anna Kreuzeder (Interim Head of the Department of Child and Adolescent Psychiatry, Clinic Hietzing), who have confirmed their willingness to cooperate in the aforementioned project.

For first contact to the participants, cooperation with the staff of the facilities is central. The data collection procedure is discussed in detail during a prior informative meeting with the treating team. The treatment team is thus given the opportunity to exclude patients from the research project internally, should this negatively influence the course of treatment or recovery of these children and adolescents or be judged as inadequate for other reasons.

In a further step, first the legal guardians and then the children and adolescents themselves are asked for their consent to participate.

3.5. Briefly outline the selection of participants as well as inclusion and exclusion criteria (if applicable: provide specific reasons for the inclusion of individuals from protected groups, such as minors, individuals who are temporarily or permanently not able to give their consent).

Underage children and adolescents are the primary target group of clown visits in psychiatric contexts, which is why the research question in the study project is aimed at this group of people. The scientific relevance of the topic is based on an existing gap in research on the effect of clown visits in the psychiatric context in Austria. The following inclusion and exclusion criteria are taken into account when selecting participants:

Children and teenagers
Inclusion criteria: (a) outpatient or inpatient psychiatric treatment at one of the cooperating facilities, (b) regular participation in clown visits, (c) age range 7 to 18 years, (d) informed consent of parent/guardian and child/adolescent.

Exclusion criteria: (a) unwillingness to participate in clown visits, (b) assessment by treating staff of negative consequences from participation in clown visits and/or data collection.

Treatment staff
Inclusion criteria: (a) responsibility for treatment (medical staff, paramedical staff, caregivers), (b) knowledge of the clinical diagnosis and treatment of children and adolescents.

The screening of mentioned criteria is carried out by qualified study personnel in discussion with the treating personnel, the legal guardians as well as the children and adolescents themselves.

3.6. Will you obtain the consent from the participants or from their legal representatives?

☒ Yes (please include information sheets and the declaration of consent)

☐ No If no, why not:

n.a.

3.7. What is the relationship (personal, social, institutional) between the participants in the study and the persons carrying out the study (e.g. student-teacher, employee-employer, etc.)? Can you guarantee that the consent to participating in the study is given voluntarily?

Institutional relations exist between the association ROTE NASEN Clowndoctors and the cooperating institutions in the psychiatric-clinical field. The association ROTE NASEN Clowndoctors will take over the logistic support in the study project. In addition, care will be taken to ensure that the clown visit takes place under routine conditions as far as possible. The clowns or other employees of the association will not be involved in any steps of data collection or evaluation.

All participants in the study (children, adolescents, treating staff) are informed verbally in advance about the study process, possible consequences, the voluntary nature of participation, and the possibility of dropping out at any time without giving reasons. This information process is carried out both with the legal guardians and with the children and adolescents themselves in an age-appropriate manner. Particular attention is paid to the rights and protection of minors, which is also the aim of the first information session with the treating staff.

3b Data protection

Please distinguish between anonymisation and pseudonymisation!
Anonymisation means to render it irreversibly impossible to associate data with a person. For this reason, anonymous and anonymised data are outside the remit of data protection laws.
Pseudonymisation means that it is possible, with the help of additional information (typically an identification key) to associate personal data with a person (see Art 4(5) General Data Protection Regulation). Pseudonymised data are within the remit of data protection laws.

3.8. What types of personal data (i.e. information that relate to an identified or identifiable natural person) will be collected?

Prior to the health-specific data collection in Phase 1, the following personal data of the children and adolescents will be requested: name, gender, date of birth, native language, email address.

Personal data is also documented in order to collect the assessment of the staff: Name, gender, age, position, time in the position at the cooperating institution, amount of working hours.

Health-specific data are also recorded as part of the survey of psychological stress measures. This includes the assessment of personal well-being as well as trait and state measurement data. Standardized questionnaires from the Department of Clinical Psychology are used for this purpose, which are classified as harmless.

3.9. How will you guarantee the anonymity of the participants?

The study is subject to the current provisions of the European Data Protection Regulation (DSGVO) as well as the respective institution-specific guidelines for handling medical data. All personal and health-related data collected over the course of the study are stored pseudonymously using a code assignment. The study staff, who coordinate the data collection on site, ensure confidential treatment of the data as well as prompt transfer of the data to the project management. All data are stored on a protected data server of the University of Vienna with exclusive access authorization by the project management. Individualized data can only be retrieved at a later date if the participants leave the study for the purpose of destroying the data or if the participants or their legal guardians request access to the data.
After completion of the study, all data will be completely anonymized and stored indefinitely on a protected data server of the University of Vienna. Contact data will be irrevocably deleted at this time.

3.10. If a complete anonymisation of personal data is not possible: How will the privacy of participants be protected?

n.a.

3.11. Will you make voice recordings or videos, or take pictures?

☐ Yes ☒ No

If yes: Will you ask participants to give their consent to the recordings, videos and pictures?

n.a.

3.12. How will you guarantee that participants can discontinue their participation in the study at any time?

Before the start of data collection, all study participants (children, adolescents, treating staff) and, in the case of minors, their legal guardians, will be informed verbally and in writing about the entire study process, the voluntary nature of study participation, and the right to drop out at any time without giving reasons. Furthermore, it is pointed out that there will be no disadvantages or consequences for the participants in case of a drop-out.

3.13. How will the data collected during the study be processed and analysed?

☐ person-related, reason:

n.a.

☒ indirectly related to the person How will the data be anonymised?

All collected data are pseudonymized in a first step. For this purpose, a code is assigned, which enables the matching of data collected more than once from the same respondents, but in any case does not allow any personalization. Access to the coding is only possible for the project management by means of a coding list. This list is stored on a protected data server at the University of Vienna.

An early termination of the study participation as well as the wish of a participant or his or her legal guardian to inspect the data are exclusive reasons for which a decoding is made possible.
One month after completion of the study, all data is completely anonymized, which excludes any personalization of the data. Note: Please note that, in consideration of data protection, we recommend to use only anonymised data.

3.14. Where, in what form and for how long will the following data be recorded and/or stored? Who can access these data?

a. Declaration of consent

Written consent forms from all participants (children, adolescents, legal guardians, treated staff) are forwarded promptly by the study staff to the project management. The project management has exclusive access to the signed declarations, which are stored in secure premises of the University of Vienna for an unlimited period of time.

b. Study data (data collected as part of the relevant study)

Completed pseudonymized questionnaires are handed over promptly by the study staff to the project management. The project management ensures that the paper-based data are stored in secure rooms at the University of Vienna. One month after completion of the study, the data will be irrevocably anonymized and it will no longer be possible to view the data for personalization purposes.

c. Case history questionnaires (if available)

n.a.

d. Human samples (e.g. blood samples, saliva samples) (if available)

The salivettes used with the saliva samples collected are temporarily stored in the institute's own refrigerators at the clinical facility where the survey is conducted. The labeling is already pseudonymized. After completion of the survey on site, the samples are promptly transported to the analytical laboratory by the study staff on a weekly basis.

3.15. If study data are *not* stored in anonymised or pseudonymised form: When is this the case and what are the reasons?

n.a.

3.16. What security measures will you take to protect the participants' data against unauthorised access? (e.g. virus protection/firewall, individual log-in and password protection, administration of permissions, encrypted systems, electronic access control system, security doors).

Pseudonymization of all collected data (questionnaires, salivettes with saliva samples) promptly after collection enables data protection and prevents access by unwanted third parties.

Salivettes are temporarily stored in the clinical, institution-owned refrigerators at the time of the Phase 1 survey, to which only the institution's staff and study personnel have access. The personnel are informed in detail about the logistical processes and are also obliged to maintain confidentiality when handling medical data.

All collected data are stored in completely anonymized form on a protected data server of the University of Vienna for an unlimited period of time and inaccessible to unauthorized persons. The data servers of the University of Vienna are password protected and can only be accessed after authorization by the Central IT Service of the University of Vienna. Only authorized and sworn representatives of domestic and/or foreign health authorities and the respective ethics committees can view the non-encrypted data, as far as this is necessary or prescribed for the verification of the proper conduct of the study. All persons who have access to the encrypted and non-encrypted data are subject to the Data Protection Regulation (DSGVO) and the Austrian adaptation regulations as amended from time to time when handling the data. Likewise, the study directors confirm with their signature that they will treat all data as well as personal information of participating persons, which they learn during their work in the study, strictly confidential.

3.17. How can participants in the study access their personal study data?

Upon request, participants or their legal representatives will be allowed to view the collected pseudonymized data up to one month after the end of the study. For this purpose, a written, informal request must be submitted to the project management, which will subsequently arrange an appointment for the inspection. The inspection will take place at the premises of the University of Vienna.

3.18. Can participants obtain information about the research results?

☒ Yes ☐ No If no, reason:

During the study phase, participants have the opportunity to contact the contact persons specified in the consent form with any questions they may have. In addition, the results are made available to the cooperating institutions in written and anonymized form. The study participants also have the opportunity to view the final report. For this purpose, an informal written interest must be expressed to the project management.

3.19. How and for how long can participants request the erasure of their study data?

A subsequent deletion of the collected pseudonymized data can be requested by the participants up to one month after the end of the study. For this purpose, an informal request must be submitted in writing to the project management. This request will also be approved in writing by the project management, which will also carry out the deletion.

3c Consequences for participants in the study

3.20. Is participating in the study associated with foreseeable risks or other foreseeable problematic side effects (e.g. pain, discomfort or violations of personal integrity)? What measures to you take to prevent and/or take care of unforeseeable/unwanted side effects?

With regard to the study procedures, no threats to the psychological or physical integrity of the participants in the sense of negative, harmful outcomes are to be expected. Any form of data collection in the planned study is to be considered harmless for the study participants, including the collection of saliva samples. Measurements are non-hazardous, non-invasive, and not associated with any pain or known risks. All devices used are approved for studies on minors. In addition, the participating children are informed about the apparatus and the nature of the measurement procedure in a child-friendly manner before the tests begin.

When answering the questionnaires, it is possible under certain circumstances that unexpected or unpleasant feelings may arise in the participants. The subjects are informed in advance that in such a case they should seek contact with the test administration or the staff treating them.

A special focus is placed on a detailed educational discussion with the medical treatment staff as well as on an age-appropriate education of the participants. In this way, the risk of negative consequences for the course of treatment due to study participation as well as the risk of involuntary participation of the children and adolescents can be excluded.

3.21. What procedures will you apply to identify, document and report on unwanted effects of participating in the study? Please describe who will take care of it and when and how this will happen, e.g. interviews and/or based on lists of questions.

In an initial informative meeting with the treating staff of the children and adolescents, they are given the opportunity to clarify internally their participation in the study with regard to possible risks for the clinical course of treatment of each child or adolescent. Emphasis will be placed on a detailed explanation of any process as well as an inspection of the study's data collection instruments. Decisions to exclude a child or adolescent by the treating staff do not need to be justified to the project team under any circumstances and will be made to the exclusion of those responsible for the project.

In the informational interview with the children and adolescents, special emphasis is placed on explaining the rights of the participants in an age-appropriate manner. The aspects of voluntariness and the right to terminate participation at any time as well as the possibility of contacting the project management at any time in case of questions or concerns are emphasized in this conversation as well as in the written version.

The data collection itself (questionnaires, saliva sample collection) takes place discreetly, but in the presence of the study staff and, in the case of saliva collection, also of a person from the treatment team. The participants are thus given the immediate opportunity to contact the study staff for clarification in the event of any difficulties arising.

3.22. Will participants receive an attendance or expense allowance (compensation for travel expenses and loss of earnings)?

Participation in the study is not associated with any costs for the subjects. Data collection will take place at the premises of the cooperating clinical institutions (phase 1) or electronically (phase 2). Legal guardians will be contacted by telephone or electronically.

Participation is calculated with a low time commitment. Monetary compensation for participation is not provided. As a small expense allowance, the association ROTE NASEN Clowndoctors provides all participants with a surprise box. In addition, the participating children and young people will receive a certificate of participation in the research project.

3.23. What advantages and/or possible benefits can participants in the study expect?

The participating children and adolescents are given an insight into the procedure of a quasi-experimental study in the field of stress research. After completion of the study, all participants are given access to the anonymized final report on the results, to which they are individually informed in writing. Attention will be paid to age-appropriate mediation.

The staff of the cooperating institutions has the opportunity to contribute to the scientific treatment of a question within their field of work. The results achieved also have practical benefits for the participating staff of the facilities. The increase in knowledge can contribute to the professional care of the target group.

3d Additional ethical aspects

3.24. Will participants be fully informed about the type, objectives and content of the study?

☒ Yes ☐ No, reason:

n.a.

Will participants be deceived?

☐ Yes ☒ No

If yes: Description and reason why it is necessary to deceive the participants:

n.a.

3.25. In your opinion, are there any other potential problems regarding participation in and implementation of the study?

According to the assessment of the project management, no serious problems can arise with regard to study participation or implementation.

3.26. What is the relation between the potential risks of participating in the study and the expected academic and social benefits?

Potential risks of the study are rated as very low. In our opinion, the scientific relevance of the topic as well as the social and health-related benefits of the study outweigh the potential risks.

3.27. Under what circumstances will the study be interrupted? Under what circumstances will the study be terminated entirely?

The project management sees no reason for the study to be terminated entirely prematurely. Due to current global restrictions in the wake of the SARS-CoV-2 spread, the planned data collection could be postponed should clown visits to collaborating facilities be restricted due to infection spread measures.<br><br>Nevertheless, the project management reserves the right to terminate the participation of certain persons during the course of the study. Reasons for this may be that the person noticeably shows no interest in the survey or cannot meet the requirements of the survey instruments, or that his or her state of health is impaired by participation. This condition is stated in writing in the information sheet for participants.

4. Other notes

none

5. Persons carrying out the study

Please list all persons working on the study:
If applicable, please distinguish between the functions of project leader (leader of the entire project), study leader (leader of a partial study) and trial leader (carrying out a study). Make sure to clearly distinguish

between these different functions and responsibilities in the information provided to participants and the declaration of consent.

Name	Institution	Function*	Qualification**
Univ.-Prof. Dr. Martina Zemp	Department of Clinical and Health Psychology, Faculty of Psychology, University of Vienna	Principal Investigator (PI)	University Professor
Prof. Dr. Urs Nater	Department of Clinical and Health Psychology, Faculty of Psychology, University of Vienna	Co-PI	University Professor
MMag.a Simone Seebacher MA	ROTE NASEN Clowndoctors	Project Coordinator	MMag, MA
Amos Friedrich, BSc	Department of Clinical and Health Psychology, Faculty of Psychology, University of Vienna	Study execution/ management	M.Sc. cand.
Lorena Holzmeier, BSc	Department of Clinical and Health Psychology, Faculty of Psychology, University of Vienna	Study execution/ management	M.Sc. cand.

* e.g. study leader, project leader, trial leader, planning, analysis, collection of data

** e.g. senior researcher, doctoral candidate, postdoctoral candidate, etc.

Are there any conflicts of interest between the researchers involved?

☐ Yes ☒ No

If yes: Complete the supplementary form on a conflict of interest and enclose it.

6. Name and signature of the applicant

Name: Prof. Dr. Martina Zemp
Institution/company: Department of Clinical Psychology of Childhood and Adolescence, Faculty of Psychology, University of Vienna
Position: University Professor

Signature of the applicant: I herewith confirm that all the information regarding the planned study provided in this application is correct and that the study will be carried out in accordance with this information and pursuant to the code of good academic practice.

I declare that I will not start the empirical part of the submitted study (recruitment, collection of data) before obtaining the vote of the Ethics Committee.

Wien, 22.3.2021

Signature of the applicant, date

6.1. Person authorised to receive notifications
